# Changes in Positive End-Expiratory Pressure Alter the Distribution of Ventilation within the Lung Immediately after Birth in Newborn Rabbits

**DOI:** 10.1371/journal.pone.0093391

**Published:** 2014-04-01

**Authors:** Marcus J. Kitchen, Melissa L. Siew, Megan J. Wallace, Andreas Fouras, Robert A. Lewis, Naoto Yagi, Kentaro Uesugi, Arjan B. te Pas, Stuart B. Hooper

**Affiliations:** 1 School of Physics, Monash Institute of Medical Research, Clayton, Victoria, Australia; 2 The Ritchie Centre, Monash Institute of Medical Research, Clayton, Victoria, Australia; 3 Department of Pediatrics, Leiden University Medical Centre, Leiden, Netherlands; 4 Department of Mechanical and Aerospace Engineering, Monash University, Clayton, Victoria, Australia; 5 Department of Medical Imaging and Radiation Science, Monash University, Clayton, Victoria, Australia; 6 Biomedical Engineering, University of Saskatchewan, Saskatoon, Canada; 7 SPring-8/Japan Synchrotron Radiation Research Institute, Sayo, Hyogo, Japan; Icahn School of Medicine at Mount Sinai, United States of America

## Abstract

Current recommendations suggest the use of positive end-expiratory pressures (PEEP) to assist very preterm infants to develop a functional residual capacity (FRC) and establish gas exchange at birth. However, maintaining a consistent PEEP is difficult and so the lungs are exposed to changing distending pressures after birth, which can affect respiratory function. Our aim was to determine how changing PEEP levels alters the distribution of ventilation within the lung. Preterm rabbit pups (28 days gestation) were delivered and mechanically ventilated with one of three strategies, whereby PEEP was changed in sequence; 0-5-10-5-0 cmH_2_O, 5-10-0-5-0 cmH_2_O or 10-5-0-10-0 cmH_2_O. Phase contrast X-ray imaging was used to analyse the distribution of ventilation in the upper left (UL), upper right (UR), lower left (LL) and lower right (LR) quadrants of the lung. Initiating ventilation with 10PEEP resulted in a uniform increase in FRC throughout the lung whereas initiating ventilation with 5PEEP or 0PEEP preferentially aerated the UR than both lower quadrants (p<0.05). Consequently, the relative distribution of incoming V_T_ was preferentially directed into the lower lobes at low PEEP, primarily due to the loss of FRC in those lobes. Following ventilation at 10PEEP, the distribution of air at end-inflation was uniform across all quadrants and remained so regardless of the PEEP level. Uniform distribution of ventilation can be achieved by initiating ventilation with a high PEEP. After the lungs have aerated, small and stepped reductions in PEEP result in more uniform changes in ventilation.

## Introduction

The lungs of premature newborns are structurally and functionally immature and so have a greater tendency to collapse than the adult lung [1]–[3]. Specifically, the lung tissue is inelastic and the distal airways lack surfactant [4], which is required to reduce surface tension and promote uniform lung aeration [5], [6]. In addition, as their chest walls are highly compliant [7], the premature newborn's ability to oppose lung recoil and maintain functional residual capacity (FRC) is reduced, which may contribute to a lower FRC [8].

Positive end-expiratory pressure (PEEP) and continuous positive airway pressure (CPAP) oppose lung atelectasis by applying an internal distending pressure on the airways, which keeps them aerated at end-expiration [9]. As a result, end-expiratory pressures improve FRC development, gas exchange, lung compliance and reduce lung injury and inflammation [9]–[11]. However, providing consistent end-expiratory pressure support in the delivery room can be difficult and changing levels often cannot be avoided. Indeed, face mask leak is common [12], [13] and assisted ventilation is often interrupted by mask repositioning, suctioning or intubation, which can rapidly decrease lung gas volumes [14]. Little is known about how these pressure changes alter the distribution of ventilation within the immature lung.

In adults with a non-uniform pattern of acute respiratory distress syndrome (ARDS), increasing PEEP levels recruits lung volume preferentially in the apical lobes, which increases compliance to a greater extent in the apical than basal lobes [15], [16]. Similar observations have been made in children with ARDS [17] and a preference for the distribution of air into apical lung regions has also been observed in vertically positioned dead newborn fetal rabbits following consecutive pressure/volume loops applied using air [18]. At birth, as the immature lung lacks surfactant [4] and incompletely clears airway liquid [19], the resulting large regional differences in lung compliance likely contributes to the heterogenous ventilation commonly observed in preterm infants. Thus, it is likely that regional differences in lung compliance, which influence the distribution of ventilation within the lung at FRC, will likely change with different PEEP levels.

We have used phase contrast X-ray imaging [9], [18]–[20] to examine how changing PEEP levels alter the distribution of ventilation within the lung in preterm rabbits ventilated from birth. We hypothesized that air will preferentially enter the apical lobes at low PEEP levels and that once apical regions have aerated, higher PEEP levels mainly affect the distribution of air towards the basal lobes of the lungs.

## Methods

### Animal Procedures

Experiments were performed in the Biomedical Imaging Centre at the SPring-8 synchrotron in Japan. All experiments were approved by Animal Ethics Committees at SPring-8 in Japan and at the School of Biomedical Science, Monash University. Pregnant near-term New Zealand white rabbits (28 days of gestation; term  =  32 days) were initially anaesthetised with propofol (Rapinovet; i.v.; 12 mg/kg bolus), intubated and then maintained by isoflourane inhalation (1.5–4%). Pups were delivered by caesarean section, sedated with pentobarbital (Nembutal; 0.1 mg, i.p.) and intubated with an endotracheal (ET) tube (18G). The ET tube was occluded to prevent spontaneous breathing before ventilation onset. Pups were positioned upright in a pre-warmed (40°C) water-filled, plethysmograph (head out) located in the path of the X-ray beam within the imaging hutch, as previously described [19], [21]. The ET tube was connected to the ventilator.

### Mechanical Ventilation

**Figure 1:**
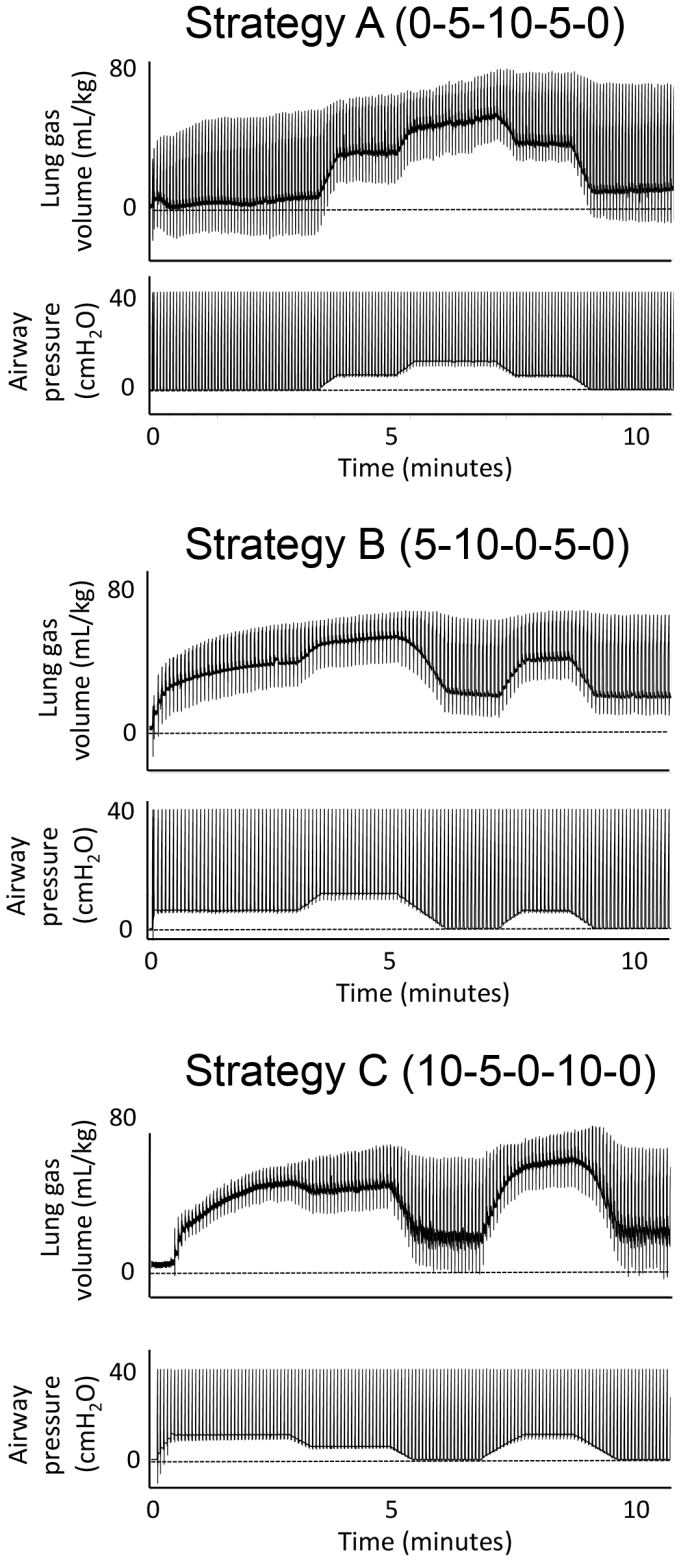
Diagram of ventilation Strategies A, B and C. Plethysmograph and airway pressure recordings of pups that were ventilated from birth using each of the PEEP Strategies A, B or C.

Pups were randomised into groups and ventilated with one of three PEEP strategies (Figure 1).

Strategy A: The initial PEEP was set at 0 cmH_2_O and was changed to 5, to 10, to 5 and to 0 cmH_2_O (0-5-10-5-0PEEP). This strategy was designed to examine the effect of gradually increasing and decreasing PEEP on lung aeration and the distribution of ventilation.

Strategy B: PEEP was initially set at 5 cmH_2_O and was then changed to 10, to 0, to 5 and to 0 cmH_2_O (5-10-0-5-0PEEP). This strategy was designed to examine the effect of PEEP recruitment (from 5 cmH_2_O) on the distribution of ventilation and the ability of 5 cmH_2_O of PEEP to re-recruit the lung following a loss of PEEP.

Strategy C: PEEP was initially set at 10 cmH_2_O and was then changed to 5, to 0 to 10 and to 0 cmH_2_O, (10-5-0-10-0PEEP). This strategy was designed to assess the ability of an initial high PEEP strategy, followed by a normal PEEP level, to aerate the lung and to assess the ability of high PEEP to re-recruit the lung following a loss of PEEP.

Phase contrast X-ray movies, found in the Supporting Information, demonstrate the effect of changing PEEP on lung aeration. Movie S1 shows a representative example of Strategy A, Movie S2 is an example of Strategy B and Movie S3 is an example of Strategy C. Pups were ventilated at each PEEP until FRC and tidal volume (V_T_) had reached a plateau; this required ∼2–3 minutes. Pups were ventilated using a SAR-830/AP (CWE Inc, Ardmore, Pennsylvania, USA) flow restrictor ventilator, which utilises a pressure-limited system and a variable bias gas flow through the ventilator circuit; in this mode, the pressure wave is triangular in shape. The peak inflation pressure (PIP) was set at 35 cmH_2_O and the PEEP was controlled by raising and lowering the expiratory tube outlet in a water-filled PEEP trap; i.e. submerging the tube 5 cm generated a PEEP of 5 cmH_2_O. Inspiratory and expiratory times were 1.0 and 1.5 sec, respectively. Changes in airway pressures were recorded digitally (Powerlab, ADInstruments; Sydney, Australia). At the conclusion of the experiment, pups were euthanized with sodium pentobarbital (Nembutal; 100 mg/kg; i.p.).

### Image Acquisition

Phase contrast images were acquired using a monochromatic X-ray beam (24 keV) with the detectors placed 2.0 m downstream from the pups (see [18]). X-rays were converted to visible light and images acquired using an electron multiplying charge-coupled device (EMCCD) camera (Hamamatsu, C9100-02). The effective pixel size was 31.82 μm and the field of view was 32(H) × 32(V) mm^2^, which captured the entire pup's chest in a single exposure. Image acquisition was synchronised with ventilation whereby inflation onset triggered the camera to acquire a sequence of six images, 300 ms apart using an exposure time of 80 ms. Three images were acquired during inspiration and three during expiration.

### Image Processing and Analysis

The first 4 lung inflations at the onset of lung ventilation and the 4 inflations immediately preceding and following a change in PEEP were used for analysis. Regional lung air volumes were calculated using the image processing technique developed by [18]. This technique utilises differences in X-ray attenuation between air and water to determine changes in gas volume within the image. Despite the images being two-dimensional (2D) projections, this technique enables very small (25 μL) changes in air volumes to be measured without having to reconstruct the three-dimensional (3D) structure using computed tomography [18].

To assess the uniformity of lung aeration, images were partitioned into quadrants using the vertebral column and the 7^th^ rib as landmarks [18]. The four quadrants were labelled upper right (UR), upper left (UL), lower right (LR) and lower left (LL) and are in reference to the image as observed. The position of the regions of interest was held constant within consecutive image by tracking the displacement of the skeleton for each pup using a cross-correlation analysis [18]. Volumetric information extracted from the lung quadrants were used to assess changes in the lung's regional functional residual capacity (FRC), V_T_ and air volumes at peak inspiratory pressure (V_PIP_). As the lung volume in each quadrant differs between quadrants and between animals within the same quadrant, lung gas volumes (FRC, V_T_ and V_PIP_) were normalised to the maximum lung gas volume obtained in each lung quadrant for each animal within a given imaging sequence. Regional dynamic lung compliance was measured as the change in lung volume in one quadrant divided by the change in airway pressure (PIP-PEEP).

### Statistical Analysis

Statistical analysis was performed using Sigmastat (Systat software Inc., USA). Results were presented as mean ± standard error of the mean (SEM) and all data were checked for normality and tested for equal variance. Changes in the distribution of ventilation at FRC, V_T_ and V_PIP_ were analysed using a 2-way repeated measures ANOVA. Changes in FRC and V_T_ in response to decreasing or increasing PEEP levels were analysed using a 2-way ANOVA. All statistical tests were followed by a Tukey post hoc test. A p<0.05 was considered statistically significant.

## Results

### Animal data

16 newborn preterm rabbit pups were ventilated from birth and the distribution of air within the lung determined using PC X-ray imaging. 6 pups were ventilated according to PEEP Strategy A, 5 according to PEEP Strategy B and 5 according to PEEP Strategy C. There were no significant differences in mean pup weight between any of the groups (p>0.05) and no pups developed a pneumothorax.

### Lung air volume distribution between quadrants

Irrespective of the strategy, the lower quadrants had significantly greater maximal lung air volumes (at end inflation) than the upper quadrants (p<0.05), which mainly reflects the size differences. The LR and LL quadrants were not significantly different from each other (20.4±1.3 mL/kg vs 17.5±1.7 mL/kg, p>0.05) and the UR and UL were also not statistically different from each other (11.7±0.6 mL/kg vs 7.7±0.6 mL/kg, p>0.05).

### Changes in gas distribution: Strategy A

#### Air distribution at FRC (Fig 2A)

Ventilation with 0PEEP initially resulted in poor aeration of the lung at FRC (<6%) in all quadrants and, although the air volume at FRC gradually increased with time, it was substantially less than with both other strategies by the end of the first ventilation period (Strategy B, Fig 3A and Strategy C, Fig 4A). Furthermore, the relative increase in air volume was unequal and was significantly greater in the UR (28.2±7.3%) than in both LL (13.7±3.0%) and LR (10.3±1.9%) quadrants (p<0.05). Subsequent ventilation at 5PEEP, improved aeration in all quadrants but the unequal distribution between quadrants at FRC remained. At 10PEEP, both upper quadrants remained better aerated at FRC than both lower quadrants (p<0.05). Following the return to 0PEEP, only the UR quadrant was significantly better aerated than both lower quadrants (UR 42.9±5.3% vs LR 29.1±5.5% and LL 26.1±5.2%, p<0.05).

**Figure 2:**
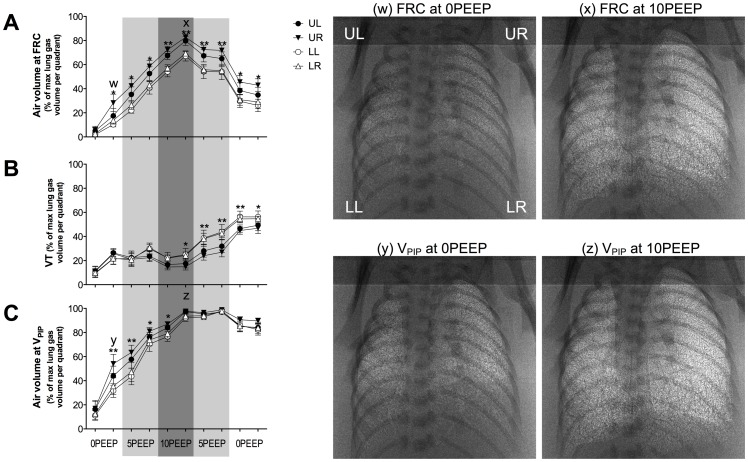
Changes in FRC, V_T_ and V_PIP_ during ventilation with Strategy A. Distribution of ventilation (A) at functional residual capacity (FRC), (B) of the incoming tidal volume (V_T_) and (C) volume at peak inflation pressure (V_PIP_) in the upper right (open triangles), upper left (closed triangles), lower right (open circles) and lower left (closed circles) regions of the lung during ventilation with Strategy A. Asterisks (*) indicate that UR quadrant is significantly different to the lower quadrants. Double asterisks (**) indicate that the upper quadrants are significantly different to the lower quadrants. Values are significantly different if p<0.05. Lower case letters (w-z) correspond to when phase contrast X-ray images of lung aeration at FRC and at V_PIP_ were acquired.

**Figure 3:**
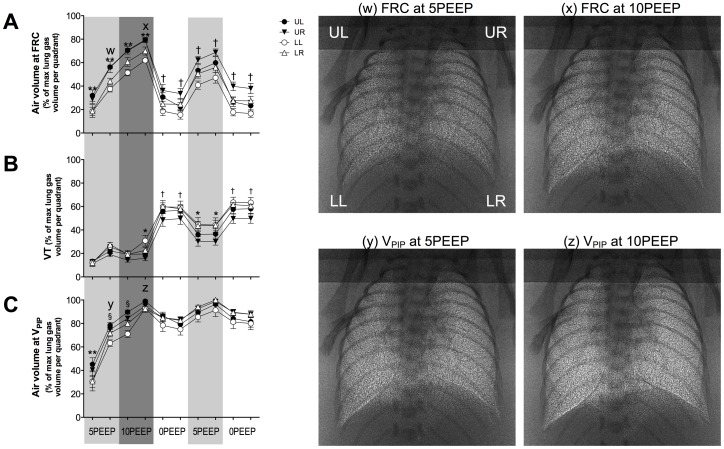
Changes in FRC, V_T_ and V_PIP_ during ventilation with Strategy B. Distribution of ventilation (A) at functional residual capacity (FRC), (B) of the incoming tidal volume (V_T_) and (C) volume at peak inflation pressure (V_PIP_) in the upper right (open triangles), upper left (closed triangles), lower right (open circles) and lower left (closed circles) regions of the lung during ventilation with Strategy B. Asterisks (*) indicate that UR quadrant is significantly different to the lower quadrants. Double asterisks (**) indicate that the upper quadrants are significantly different to the lower quadrants. Daggers (†) indicate that UR quadrant is significantly different to all other quadrants. Section symbol (§) indicates that the LL quadrant is significantly different to all other quadrants. Values are significantly different if p<0.05. Lower case letters (w-z) correspond to when phase contrast X-ray images of lung aeration at FRC and at V_PIP_ were acquired.

**Figure 4:**
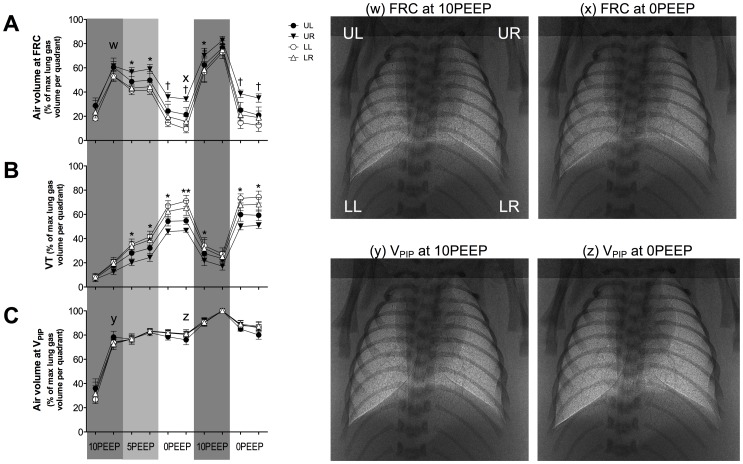
Changes in FRC, V_T_ and V_PIP_ during ventilation with Strategy C. Distribution of ventilation (A) at functional residual capacity (FRC), (B) of the incoming tidal volume (V_T_) and (C) volume at peak inflation pressure (V_PIP_) in the upper right (open triangles), upper left (closed triangles), lower right (open circles) and lower left (closed circles) regions of the lung during ventilation with Strategy C. Asterisks (*) indicate that UR quadrant is significantly different to the lower quadrants. Double asterisks (**) indicate that the upper quadrants are significantly different to the lower quadrants. Daggers (†) indicate that UR quadrant is different to all other quadrants. Values are significantly different if p<0.05. Lower case letters (w-z) correspond to when phase contrast X-ray images of lung aeration at FRC and at V_PIP_ were acquired.

#### Distribution of incoming V_T_ (Fig 2B)

Following initial lung aeration with 0PEEP, ventilation with 5PEEP and then with 10PEEP did not alter the size of the incoming V_T_, which contributed to 20–30% of total lung air volumes at end-inflation (V_PIP_). However, by the end of the 10PEEP period, the lower quadrants started to receive a significantly (p<0.05) greater proportion of this volume than the upper quadrants. With the onset of subsequent 5PEEP ventilation, both lower quadrants persisted in receiving a significantly larger proportion of the incoming V_T_ than both upper quadrants (∼43% vs ∼30%; p<0.05). This non-uniform distribution of incoming V_T_ persisted during the subsequent 5PEEP and 0PEEP periods, despite a significant increase in V_T_.

#### Air distribution at end-inflation (V_PIP_) (Fig 2c)

At the initiation of ventilation with 0PEEP, the distribution of air in the lung at V_PIP_ was preferentially directed towards both upper quadrants. By the end of the 0PEEP period, the proportion of air in the upper quadrants at V_PIP_ (range 43–54% of maximal lung gas volume) was significantly greater than the lower quadrants (∼33% of maximal lung gas volume; p<0.05). During the subsequent 10PEEP ventilation period, when the lung had fully aerated, all quadrants were similarly ventilated at V_PIP_ (>90%, p>0.05) and remained so for the rest of the experiment.

### Changes in ventilation distribution: Strategy B

#### Air distribution at FRC (Fig 3A)

During ventilation at 5PEEP and then at 10PEEP, the distribution of air in the lungs at FRC was greater in both upper than in both lower quadrants (p<0.05). Reducing PEEP to 0PEEP increased the air volumes in the UR quadrant at FRC and reduced the amount in the LL quadrant (UR 36.3±5.1% and UL 30.6±3.6% vs LL 18.7±3.9% and LR 24.8±5.0%; p<0.05). This non-uniform distribution of air in the lungs at FRC persisted during the remainder of the experimental period; relative air volumes in the UR and LL quadrants at FRC remained greater and lower, respectively, than the other quadrants (p<0.05).

#### Distribution of incoming V_T_ (Fig 3B)

Initially at 5PEEP, the distribution of incoming air with each inflation was relatively even across all quadrants, increasing from ∼10% to 30% of total volume at end-inspiration. However, following ventilation at 10PEEP, decreasing the PEEP to 0PEEP resulted in a significantly smaller proportion of the V_T_ entering the UR quadrant (p<0.05), compared with the other quadrants, despite the UR quadrant having the highest proportional air volume at FRC. This indicates that some airway closure and gas trapping may have resulted from the sudden reduction in PEEP. Importantly, subsequent ventilation at 5PEEP did not alter this pattern, with the majority of incoming V_T_ being distributed towards the lower quadrants (∼44%; p<0.05) rather than upper quadrants (∼30%).

#### Air distribution at V_PIP_ (Fig 3C)

During the initial 5PEEP and 10PEEP recruitment periods, the distribution of air in the lung at V_PIP_ predominantly occurred in the upper quadrants, whereas the LL quadrant aerated the least (p<0.05). Following ventilation at 10PEEP, all quadrants were similarly aerated at V_PIP_ and subsequent PEEP changes maintained this uniform distribution of air (p>0.05).

### Changes in ventilation distribution: Strategy C

#### Air distribution at FRC (Fig 4A)

Initiating ventilation with 10PEEP rapidly increased FRC and this air was uniformly distributed across all 4 quadrants of the lung (p<0.05). Decreasing the PEEP to 5PEEP, significantly decreased relative air volumes at FRC within the lower quadrants compared with the UR quadrant, which did not change (p<0.05). When PEEP was decreased further to 0PEEP, the air volume at FRC in all quadrants rapidly decreased, but the retention of air in the UR quadrant was better than in all other quadrants (p<0.05). This pattern of non-uniform air distribution at FRC was abolished by subsequent ventilation with 10PEEP, but returned during the second period of ventilation with 0PEEP.

#### Distribution of incoming V_T_ (Fig 4B)

Initiating ventilation at birth with 10PEEP resulted in a relatively uniform distribution of the V_T_ across all quadrants (p>0.05). Reducing PEEP to 5PEEP increased V_T_ in all quadrants, but there was less V_T_ entering the UR compared to the lower quadrants (p<0.05); the UL was not significantly different to any quadrant (p>0.05). This non-uniform pattern of incoming V_T_ distribution was also observed during both 0PEEP ventilation periods but was not observed at the end of the second 10PEEP ventilation period. During this time, the distribution of V_T_ was again relatively uniform across all quadrants (p>0.05).

#### Air distribution at V_PIP_ (Fig 4C)

Ventilation with 10PEEP rapidly increased lung air volumes at V_PIP_, which were remarkably uniform across all 4 quadrants. Importantly, this uniform distribution of air between quadrants at V_PIP_ persisted even during both periods of ventilation with 0PEEP, indicating that the initial ventilation with 10PEEP has persisting benefits on the uniformity of ventilation at V_PIP_.

### V_T_ and FRC changes in response to decreasing PEEP in a ventilated lung

**Figure 5:**
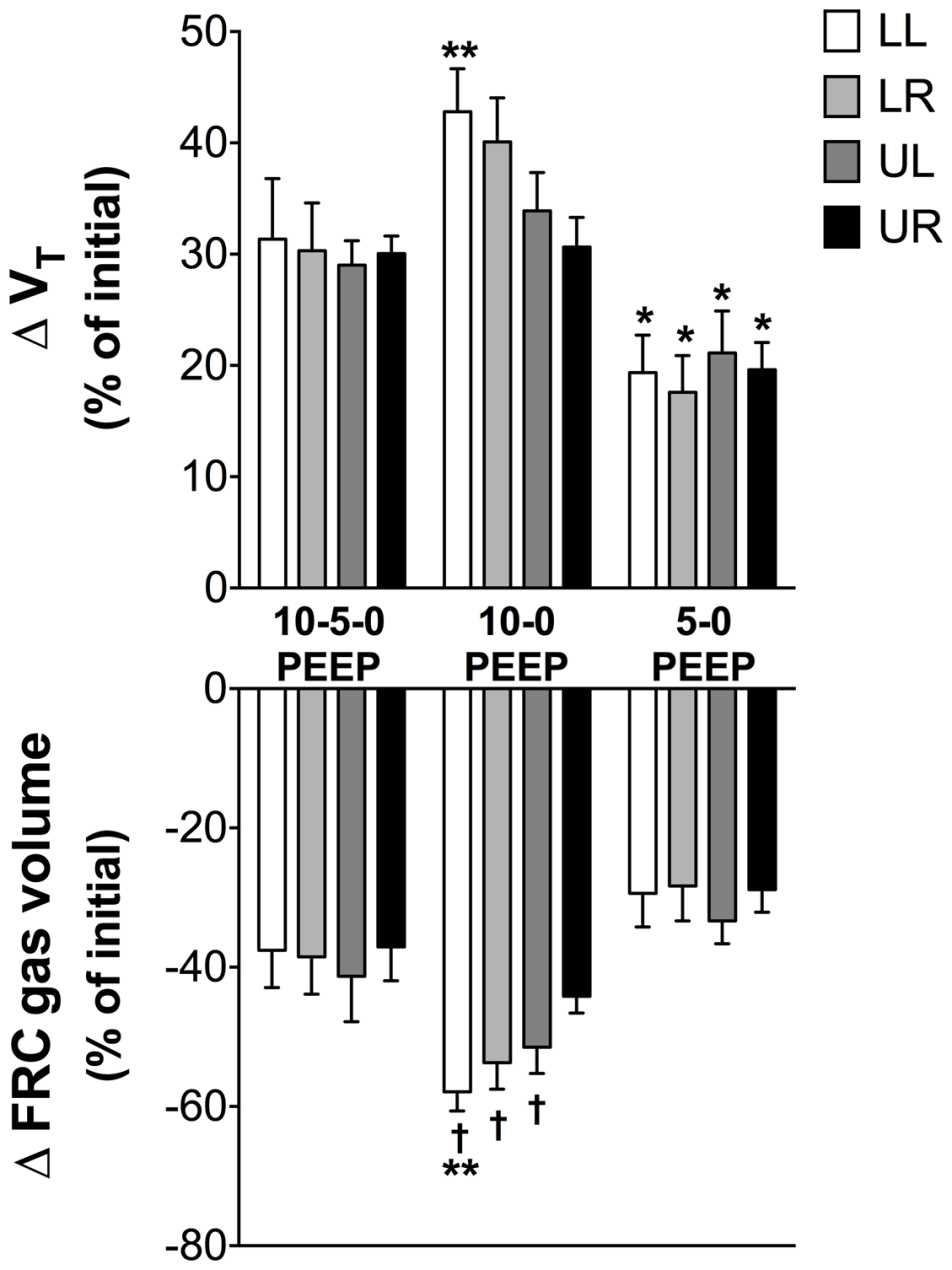
Changes in V_T_ and FRC during decreasing PEEP. The increase in tidal volume (V_T_) and decrease in functional residual capacity (FRC) when positive end-expiratory pressure (PEEP) is decreased in 5 cmH_2_O stepwise decrements (10-5-0PEEP), large steps (10-0PEEP) and from a small step (5-0PEEP) in the ventilated lung. Asterisks (*) indicate that values during 5-0PEEP are significantly different to their corresponding quadrant during 10-5-0PEEP and 10-0PEEP. Double asterisks (**) indicate that values during 10-5-0PEEP are significantly different to their corresponding quadrant during 10-0PEEP. Daggers (†) indicate that values during 5-0PEEP are significantly different to their corresponding quadrant during 10-0PEEP. Values are significantly different if p<0.05. There was no significant difference between quadrants within any group.

**Figure 6:**
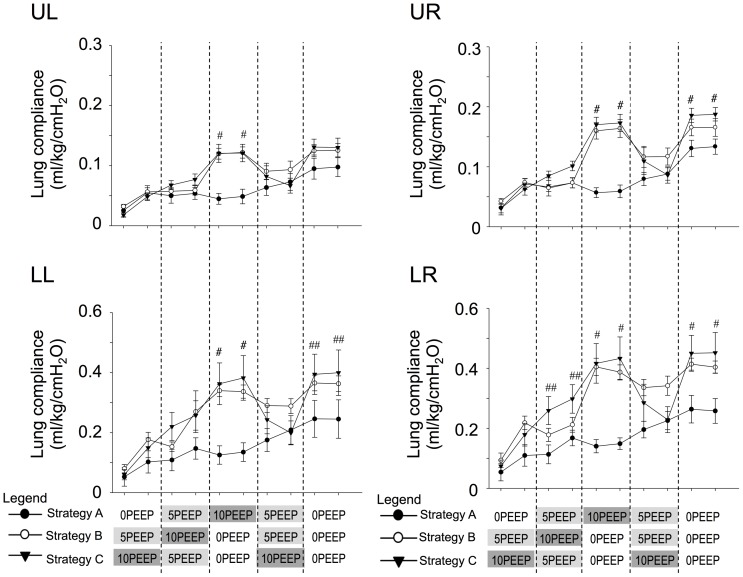
Changes in lung compliance in ventilation strategies A, B and C. Lung compliance of each quadrant (upper left (UL), upper right (UR), lower left (LL) and lower right (LR)) compared between Strategy A (closed circles), Strategy B (open circles) and Strategy C (closed triangles). The x-axis demonstrates the timing and sequence of PEEP change for each strategy. Hashes (#) indicate that Strategy A was significantly different to Strategy B and C. Double hashes (##) indicate that Strategy A was different only to Strategy C. Values are significantly different if p<0.05.

During ventilation strategies A and B, decreasing the PEEP by 5 cmH_2_O steps to 0PEEP, (ie from 10 to 5 to 0PEEP during Strategy A or from 5 to 0PEEP during Strategy B) caused a small reduction in FRC and an increase in V_T_, with the relative changes in both V_T_ and FRC being similar in all quadrants (Fig 5). In contrast, a large and continuous decrease in PEEP (ie from 10 to 0PEEP during Strategy C), resulted in changes in V_T_ and FRC that were not evenly distributed across all quadrants. Indeed, compared to PEEP reductions of only 5cmH_2_O, continuous PEEP reductions of 10cmH_2_O caused larger reductions in FRC and increases in V_T_ in both lower quadrants (p<0.05; Fig. 5). For instance in the LL quadrant the increase in V_T_ was markedly greater following the change from 10 to 0PEEP compared with the 10-5-0PEEP change (42.8±3.9% vs 31.1±5.5%, p<0.05, Fig. 6A).

### Changes in regional dynamic lung compliance (C_L_)

During all PEEP strategies, dynamic lung compliance was higher in the lower than in the upper quadrants, primarily due to size differences between quadrants, and increased with increasing lung aeration. However, this increase was markedly greater in pups that commenced ventilation with PEEP. The gradual time-related increase in dynamic lung compliance for all strategies was associated with both increasing and decreasing PEEP levels (Fig 6). Initiating ventilation with either 5 or 10PEEP resulted in much greater lung compliances, particularly in the lower lobes, irrespective of the PEEP level. Indeed, at the midway point of the experiment, when pups in strategy A were ventilated with 10PEEP and pups in both other strategies were ventilated with 0PEEP, dynamic compliances were markedly higher in all quadrants in pups ventilated with Strategies B and C, compared to Strategy A. Similarly, during the last ventilation phase, when all pups were ventilated with 0PEEP, dynamic lung compliances were higher if ventilation commenced with PEEP, particularly within both lower quadrants.

## Discussion

Our findings indicate that ventilating preterm rabbits with different and changing PEEP levels markedly effects the distribution of ventilation within the lung and on lung compliance during the immediate newborn period. We have used the quantitative capability [18] and high spatial resolution of phase contrast X-ray imaging to measure the distribution of air in the lung at both FRC and end-inspiration (V_PIP_) as well as the relative distribution of the incoming V_T_ with each inflation. We found that, in preterm rabbit pups, changes in PEEP rapidly changed the distribution of air in the lung at FRC and the relative spatial distribution of the incoming V_T_. Initiating ventilation at birth with 0PEEP, not only resulted in the lowest air volumes in all quadrants, the relative distribution of air between quadrants was not uniform, resulting in a significantly greater distribution of air towards the upper compared to the lower quadrants at FRC. Importantly, this unequal distribution of air at FRC persisted throughout the experiment even after subsequent ventilation at 5 and 10PEEP. Similarly, although initiating ventilation with 5PEEP increased lung air volumes compared to 0PEEP, the relative distribution of air was again unequal, being significantly greater in the upper compared to the lower quadrants, and persisted throughout the experimental period, even after ventilation with 10PEEP. In contrast, initiating ventilation with 10PEEP resulted in rapid FRC accumulation, which was uniformly distributed across all quadrants at both FRC and V_PIP_. Although subsequent ventilation with 5PEEP and 0PEEP resulted in the preferential distribution of air into the upper quadrants at FRC, the distribution of air at V_PIP_ remained remarkably uniform across all quadrants. As a result, during ventilation with 5 and 0PEEP in this group, the incoming V_T_ was preferentially directed into the lower lung quadrants. These data indicate that initiating ventilation at birth with higher PEEP levels has effects on the distribution of ventilation in the lung at both FRC and V_PIP_ that temporarily persist even at different subsequent PEEP levels.

The immature lung is prone to collapse at end-expiration because of its low tissue compliance, high surface tension and a highly compliant chest wall. PEEP or CPAP can prevent atelectasis by acting as an internal “splint” to promote FRC maintenance (see [21]–[24]. Our results also demonstrate that initiating ventilation with high PEEP levels (i.e. 10PEEP) promoted uniform air distribution at both FRC and V_PIP_ (Fig. 4A) whereas lower PEEPs demonstrated a more non-uniform air distribution, with lower quadrants less ventilated than upper quadrants (Fig. 2A and 3A). It is possible that the momentum of deflating lung tissue during expiration influences the distribution of air in the lung at FRC. As momentum is the product of an object's mass and velocity, the larger mass of lung tissue in the lower quadrants may generate greater momentum during expiration and be more prone to collapse than the upper quadrants, which have a smaller mass. Thus, medium PEEP levels (5PEEP) are able to oppose lung collapse in the upper lung quadrants, which have a smaller mass, whereas higher PEEP levels are required to oppose lung collapse in regions with a greater mass. Alternatively, regional differences in chest wall compliance, with the upper chest being less compliant, may account for the regional differences in FRC across the lung.

The persisting effects that the initial PEEP level had on the distribution of air within the lung at FRC during the experiment, despite subsequent changes in PEEP, are difficult to explain. When ventilation was initiated with 0 or 5PEEP, the non-uniform distribution of air at FRC persisted at all PEEP levels throughout the experiment, even during ventilation with 10PEEP. However, when ventilation was initiated with 10PEEP, the distribution of air at FRC was uniform and although the uniformity decreased with decreasing PEEP, it was restored by ventilation with 10PEEP. This indicates that the initial distribution of lung aeration may define the subsequent mechanical behaviour of localised lung regions, with regions that do not initially aerate persisting with lower compliances and lower air volumes at FRC no matter what the subsequent PEEP setting. Further studies are required to determine whether this effect PEEP extends for longer than the duration of this experiment.

In contrast to many studies that investigate the distribution of lung ventilation in supine patients [15]–[17], [25], [26], our study imaged newborn rabbit pups positioned upright in a water-filled plethysmograph. It is possible that the upright positioning of the pups during ventilation contributed to the greater distribution of air in the upper compared to the lower quadrants, although we would expect this to have been more apparent if the pups were supine due to the effect of the abdominal contents pushing the diaphragm. In this experiment, the overall influence of gravity on the lung is likely to be minimal as the pups were ventilated whilst suspended in a water-filled plethysmograph. This alleviates the weight of the more vertical lungs regions and the heart on the lower regions thus reducing the dependent/non-dependent lung effect. It also removes the influence of the abdominal contents and the upward displacement of the diaphragm on the lung that would occur if the pup were positioned horizontally. As such, the position of the pup is not expected to significantly influence the distribution of ventilation. As neonates are normally positioned supine or prone, the non-uniform ventilation distributions we observed may exist and be amplified in infants. Ventilation heterogeneity would be expected due to the effects of gravity between non-dependent and dependent lung regions [17], [25] and the pressure applied by abdominal contents on the diaphragm, which displaces it into the thorax. Ventilation inhomogeneity has been observed in ventilated infants lying supine, prone and quarter prone, but the authors suggest that ventilation distribution is minimally impacted by gravity [26].

Previous studies have cautioned against the use of high PEEP levels because it can reduce pulmonary blood flow (PBF) [27], [28], overexpand alveoli and increase the risk of lung injury [29]. As such, PEEP levels as high as 10 cmH_2_O are not recommended for neonates, but in those studies, the newborn lungs were aerated before the high PEEP level was applied. Thus, it is not known whether high PEEP levels during lung aeration at birth cause similar effects. A recent study has shown that an initial sustained inflation of ∼1 min (to 35 cmH_2_O) induces a greater increase in PBF than conventional ventilation [30]. This indicates that a sustained increase in airway pressure during lung aeration does not have the same negative impact on PBF as it does once the lung has aerated. In our study, none of the rabbit pups developed a pneumothorax, despite appearing maximally aerated at V_PIP_ during ventilation with 10PEEP (see Movies S1–S3, during the period of 10PEEP ventilation). More specifically, when ventilation commenced with 10PEEP, the lungs did not maximally aerate at V_PIP_ during the initial 10PEEP period. This suggests that the lungs were not over-expanded, either globally or in any one region by initiating ventilation with a temporary period of 10PEEP. Furthermore, a short period of ventilation with 10PEEP at birth was found to confer considerable benefit resulting in a remarkably uniform distribution of air across the lung during subsequent ventilation. While there are more familiar methods to promote uniform lung ventilation at birth, such as sustained inflations [23] and prophylactic surfactant [5], initial ventilation with a high PEEP level may be a more practical alternative.

There are many studies demonstrating multiple advantages of PEEP/CPAP on lung aeration and gas exchange in newborns, however, it is difficult to deliver these pressures uninterrupted in the delivery room. Not all positive pressure ventilation devices deliver a set PEEP reliably [31], [32] and face mask leak is common [33] and if large (>60%) substantially reduces the effective PEEP level [12]. Our results suggest that if the loss of PEEP is gradual (i.e. 10-5-0PEEP), or if the reduction in PEEP is small (i.e. 5-0PEEP), the changes in V_T_ and FRC are relatively uniform throughout the lung. (Fig. 5). However, large and continuous reductions in PEEP (i.e. 10-0PEEP) caused large changes in V_T_ and FRC predominantly within in the lower lung quadrants (Fig. 5). In the delivery room, respiratory support is often interrupted to reposition the facemask, perform suction, to change mask size, to change the interface for transport and during transfer of the infant to a transportation crib. Previous studies in preterm infants at 7 days of postnatal age have shown that such a rapid reduction in PEEP reduces FRC by as much as 30% within a minute of removing the PEEP [34]. Based on our results, sudden and large losses in PEEP would alter the distribution of ventilation within the lungs, causing marked reductions in FRC and greater V_T_ changes in the dependent lobes thereby increasing the risk of shear stress injury in these regions. While such interruptions in respiratory support are often necessary, the risks could be minimised by 1) reducing the PEEP gradually and 2) reducing the size of the dependent lung by placing the infant on its side, preferably the left.

It is unclear why the degree of continuous PEEP decrease affected the distribution of air at FRC and the incoming V_T_, as the total changes in lung air volumes at 0PEEP were the same between 10-0PEEP and 10-5-0PEEP (data not shown). It is unlikely to be due to the rate of PEEP decrease, because the maximum rate of PEEP decrease was ∼1 cmH_2_O per inflation, such that a 5 cmH_2_O decrease required ∼5 inflations and a 10 cmH_2_O decrease required ∼10 inflations. It is possible that a large continuous decrease in PEEP generates momentum within the lung tissue, particularly in the lower quadrants with larger masses, resulting in larger decreases in FRC with each expiration. However, when the decrease in PEEP was interrupted by a period of ventilation at 5PEEP, the distribution of ventilation between quadrants remained relatively even despite the reduction to 0PEEP, again indicating that previous PEEP history can impact on the distribution of ventilation within the lung.

It is likely that the lower lung quadrants had greater volume changes in response to changes in PEEP because these lung regions are larger and are thus more compliant (Fig. 6). It may appear counter intuitive that dynamic lung compliance in all quadrants gradually increased during ventilation with both an increasing (Strategy A) and decreasing (Strategy B) PEEP strategy. Increasing PEEP and PEEP recruitment strategies are known to increase lung compliance by allowing tidal expansion to occur within a more compliant region of the pressure volume curve [11], [35]. However, these pups were premature and surfactant deficient and the initial dynamic compliance measurements are dominated by the presence of airway liquid, which increases airway resistance ∼100 fold [20]. Thus, the gradual increase in lung compliance mainly reflects the gradual increase in lung aeration. As such, our findings indicate that the presence of airway liquid is the dominant determinant of dynamic lung compliance during lung aeration. However, following complete lung aeration (usually after ventilation with 10PEEP), increasing PEEP from 0PEEP (Strategies B & C) mostly reduced measures of dynamic lung compliance, whereas decreasing PEEP to 0PEEP increased dynamic lung compliance. Furthermore, during the final 0PEEP ventilation period in all strategies, regional lung compliance, particularly in the lower quadrants, was markedly greater if ventilation had commenced with PEEP (either 5 or 10 cmH_2_O) compared with 0PEEP. Although the mechanism is unknown, our study demonstrates that initiating ventilation with PEEP effects subsequent ventilation. These effects were observed for 10 minutes in our experiment but, as lung aeration continues to increase, these affects will likely persist beyond the experimental period.

After the lungs had fully aerated (ie after ventilation with 10PEEP in all strategies), as all pups were ventilated with a set PIP of 35 cmH_2_O, the air volumes and the distribution of air at V_PIP_ remained relatively stable at all subsequent PEEP levels. Thus, although V_PIP_ tended to decrease at 0PEEP, the increase and decrease in V_T_ associated with decreases and increases in PEEP, respectively, mainly resulted from decreases and increases in FRC. This indicates that the effect of PEEP on the pressure-volume (P-V) relationship of the aerating lung immediately after birth is substantially different than it is following complete lung aeration. We found that altering the PEEP level had little or no effect on lung air volumes at V_PIP_, resulting in a lower V_T_ when the FRC increased in response to a higher PEEP. How this effects CO_2_ clearance is unclear as, although V_T_ is reduced which would normally increase CO_2_ retention, our previous study has shown that V_PIP_ is a primary determinant of CO_2_ clearance during lung aeration [36].

At birth, as the lungs make their transition from a liquid-filled to an air-filled organ, changes in PEEP have a marked influence on the distribution of ventilation. Our results demonstrate that when initiating ventilation at birth, the uniformity of ventilation depends on the level of PEEP, with higher PEEPs promoting more uniform ventilation. As such, it is possible that starting infant resuscitation with higher PEEP levels and then gradually reducing the PEEP to currently recommended levels after a few minutes may increase the distribution of ventilation in preterm infants. Furthermore, after the lungs were fully aerated, larger continuous changes in PEEP reduced ventilation within the lower lung quadrants to a greater extent than in the upper lung quadrants. Since providing consistent assisted ventilation at birth is difficult, due to face mask leak or the need to temporarily remove pressure support, it is important to acknowledge that changes in PEEP, even brief, are not without impact on lung volumes and ventilation distribution that can persist. These effects may impair gas exchange or increase the risk of lung damage.

## Supporting Information

Movie S1 **Phase contrast X-ray movie of a preterm rabbit pup ventilated with Strategy A (0-5-10-5-10PEEP).**
(AVI)

Movie S2 **Phase contrast X-ray movie of a preterm rabbit pup ventilated with Strategy B (5-10-0-5-0PEEP).**
(AVI)

Movie S3 **Phase contrast X-ray movie of a preterm rabbit pup ventilated with Strategy C (10-5-0-10-0PEEP).**
(AVI)
